# Electrolyte Imbalance at Admission Does Not Predict the Length of Stay or Mortality in Dengue-Infected Patients

**DOI:** 10.7759/cureus.10419

**Published:** 2020-09-13

**Authors:** Fazal U Rehman, Syed Furrukh Omair, Fatima Memon, Imrana Amin, Bakhtawar J Rind, Sumera Aziz

**Affiliations:** 1 Medicine, Aga Khan University Hospital, Karachi, PAK; 2 Internal Medicine, Dow University of Health Sciences, Karachi, PAK; 3 Medicine, Patel Hospital, Karachi, PAK; 4 Medicine, Jam Ghulam Qadir Hospital Hub District Lasbela, Quetta, PAK; 5 Community Health Sciences, Aga Khan University hospital, Karachi, PAK

**Keywords:** dengue virus, length of stay, mortality, electrolyte imbalance

## Abstract

Background

A pattern of both clinical and biochemical abnormalities is associated with dengue virus infection (DVI). Among the various DVI-related biochemical defects, electrolyte imbalance is one that can alter the morbidity and mortality among patients. However, there is a dearth of evidence to assess the relationship between electrolyte imbalance and the length of stay or mortality in dengue-infected patients in Pakistan. In the current study, we aimed to investigate the association between electrolyte imbalance at the time of admission and the length of stay and mortality among dengue-infected patients.

Methods

We conducted a retrospective study at a large tertiary care hospital from November 2018 to November 2019. All patients with known chronic diseases and coinfections or those who were taking diuretics therapies or angiotensin-converting enzyme inhibitors were excluded. Our main exposure of interest was electrolytes imbalance and the outcome measure was the length of stay and mortality.

Results

A total of 1,008 dengue patients were enrolled with a mean length of stay of 2.56 days. Around 29.3% had hyponatremia and 23.2% had hypokalemia at the time of admission, and 21.9% of patients had a stay beyond three days. In multivariable analysis, hyponatremia [adjusted odds ratios (aOR) = 1.29; 95% confidence interval (CI): 0.59-2.84] and hypokalemia (aOR = 2.36; 95% CI: 0.91-6.10) were not found to be associated with the length of stay. However, patients with high troponin levels at admission had a prolonged stay beyond three days (aOR = 5.74; 95% CI: 2.34-14.11). There was a statistically significant association of creatinine levels (aOR = 14.74; 95% CI: 4.19-15.85) and diabetes mellitus (DM) (aOR = 4.36; 95% CI: 1.21-15.74) with mortality after controlling for potential confounders.

Conclusion

Electrolyte imbalance at admission is not a predictor of length of stay or fatalities in the hospital among patients with DVI. However, troponin levels at admission can increase hospitalization days whereas DM and renal injury have been found to worsen mortality rates.

## Introduction

The considerable surge in dengue virus infections (DVI) in recent years has made it the most swiftly spreading arthropod-borne viral disease caused by one of the four serotypes of dengue viruses from the *Flaviviridae* family. Originating in Southeast Asia, it has spread to South America and Africa over the years and is now endemic in over 125 countries [1,2]. According to the World Health Organization (WHO) estimates, 50 million dengue infections occur annually, and more than 2.5 billion people who are living in endemic areas are at risk of dengue infection [3]. Southeast Asia accounts for more than 75% of the global burden of the disease [3,4].

Pakistan has been considered dengue-endemic since 1994, and the disease has become a public health problem since 2006, resulting in around 147,200 cases and over 800 deaths from 1995 to 2019 [5]. Recently, a massive dengue outbreak was reported in Pakistan, beginning in July 2019, with a total of 47,120 cases of dengue fever reported between July and November 2019 [6]. Dengue is primarily a self-limiting illness that is characterized mainly by fever, headache, joint aches, rash, nausea, vomiting, and various biochemical abnormalities [7]. A pattern of these abnormalities ranges from hyponatremia and hypokalemia to an accumulation of blood urea nitrogen and decreased creatinine clearance [8]. Treatment of dengue is mainly supportive and needs appropriate and watchful fluid therapy, specific organ support, and rectification of metabolic abnormalities [9].

Early screening for electrolyte imbalances at admission is recommended in various studies to find out if the patient is at a higher risk of complications, to avoid the serious consequences associated with dengue infection, and to initiate appropriate fluid therapy [10]. Clinical observations have shown that altered levels of the electrolytes such as sodium and potassium might affect the length of stay and mortality in dengue-infected patients. However, there is a scarcity of studies affirming the same observation. Therefore, we conducted this study to assess the effect of electrolyte imbalances on the length of stay and mortality in dengue-infected patients. The findings of the study will help in framing an appropriate management plan in preventing malicious consequences associated with this disease.

## Materials and methods

Study design and setting

We conducted a retrospective study among hospitalized patients diagnosed with dengue infection from November 2018 to November 2019. The study was conducted at a 700-bedded tertiary care hospital in Karachi, Pakistan, with an annual inpatient census of 6,000-8,000 approximately.

Study subjects

All patients of 18 years of age or older with a clinical diagnosis of DVI admitted to the hospital were enrolled in the study. The lab results were reviewed through the online portal and patient care services. We included only those patients with a positive dengue antigen NS-1 or those with a dengue diagnosis confirmed by an anti-dengue immunoglobulin M (IgM) assay using an antibody-capture enzyme-linked immunosorbent assay (PanBio, Brisbane, Australia).

Exclusion criteria encompassed patients with pre-existing cardiac, renal, and hepatic dysfunctions [chronic kidney disease (CKD) and chronic liver disease]. In addition, those patients who were on any medicine affecting electrolytes, such as diuretics, angiotensin-converting enzyme inhibitors, or antidepressants were also excluded. We also excluded patients with an established dual infection such as dengue with malaria or dengue with *Salmonella typhi*.

Study variables and outcome measure

The standardized extraction of demographic and clinical data from the electronic medical records was performed by trained data abstractors. Clinical information abstracted included age (years), gender, comorbidities [diabetes mellitus (DM), hypertension (HTN), ischemic heart disease (IHD), and CKD] and length of hospital stay. For the purpose of analysis, we categorized continuous variables such as patient’s age into two categories: <35 years and ≥35 years. Sodium level of <135 meq/L was labeled as hyponatremia and potassium of <3.5 meq/L was labeled as hypokalemia. Likewise, troponin above 0.04 ng/ml was taken as elevated and serum creatinine was categorized into a binary variable: <1.3 mg/dl and ≥1.3 mg/dl. Other categorical variables such as DM, HTN, and pleural effusion were marked as yes if present and no if absent. The main outcome of interest was the length of stay, which was categorized into <3 days and ≥3 days, and the secondary outcome was the mortality rate (died in the hospital/not).

Statistical analysis

We performed descriptive statistics and regressions to analyze the data. We generated frequencies and percentages of independent and dependent variables. The associations between the categorical independent variables of interest and the dichotomous outcome variable were tested by using a chi-square test (χ2). We performed a univariable analysis to examine the determinants of the length of stay and mortality. Additionally, we performed multivariable regression using variables that were significant with an alpha of 0.05 levels in binary analysis. We reported crude and adjusted odds ratios (aOR) and reported 95% confidence intervals (CI) after adjusting for important confounders such as age, gender, troponin levels, platelet count, and comorbidities such as DM and HTN. P-values of less than 0.05 were considered statistically significant. We used SPSS Statistics software for Windows version 13.0 (IBM, Armonk, NY).

Ethical consideration

The study was reviewed and approved by the Ethical Review Committee of the Aga Khan University Hospital, Karachi, and was exempted from written informed consent.

## Results

Sociodemographic and clinical characteristics of patients admitted with dengue fever

We found that around more than half of the patients (55.4%) were older than 35 years; 68.4% were males with a mean length of stay of 2.58 days as shown in Table 1. Moreover, 10.8% of the patients were diabetic and 12.4% were hypertensive. Regarding levels of sodium at the time of admission, slightly lower than one-third of the patients (29.3%) were diagnosed with hyponatremia, 23.2% were hypokalemic, and a similar proportion of the patients had lower levels of HCO_3_. One-third of the patients (31.3%) had higher levels of troponin and 28.9% had a platelet count of less than 40,000. On the other hand, only 9% were diagnosed with pleural effusion, and 1.2% with acute cholecystitis, and cardiac pathologies like valvular lesions or wall motion abnormalities on echocardiography. With respect to the mode of discharge, most of the patients (93%) were discharged; however, 1.6% died in the hospital (Table 1).

**Table 1:** Sociodemographic and clinical characteristics of dengue patients admitted to a tertiary care hospital (n=1,008) *P-values are generated using the chi-square test OR: odds ratio; NA: not applicable; CI: confidence interval; LAMA: left against medical advice

			Length of stay					Crude estimates		
	Total		≤3 days		>3 days		P-value*	OR	95% CI	
	N	%	N	%	N	%				
Age, years										
≤35	450	44.6	359	45.6	91	41.2	0.24	1		
>35	558	55.4	428	54.4	130	58.8		1.19	0.88	1.62
Gender										
Male	689	68.4	549	69.8	140	63.3	0.07	1		
Female	319	31.6	238	30.2	81	36.7		1.34	0.97	1.83
Diabetes mellitus										
No	899	89.2	710	90.2	189	85.5	0.04	1		
Yes	109	10.8	77	9.8	32	14.5		1.56	1.01	2.43
Hypertension										
No	883	12.4	716	91	167	75.6	<0.001	1		
Yes	125	87.6	71	9	54	24.4		3.26	2.2	4.83
Ischemic heart disease										
No	975	96.7	770	97.8	205	92.8	<0.001	1		
Yes	33	3.3	17	2.2	16	7.2		3.54	1.76	7.12
Chronic kidney disease										
No	995	98.7	780	99.1	251	97.3	0.03	1		
Yes	13	1.3	7	0.9	6	2.7		3.11	1.03	9.35
Sodium levels, meq/L										
≥135	683	70.7	546	72.3	137	64.9	0.03	1		
<135	283	29.3	209	27.7	74	35.1		1.41	1.02	1.95
Potassium levels, meq/L										
≥3.5	741	76.7	587	77.7	154	73	0.15	1		
<3.5	225	23.2	168	22.3	57	27		1.29	0.91	1.83
Platelets										
>150,000	151	15	111	14.1	40	18.1		1		
40,000–150,000	566	56.2	441	56	125	56.6	0.22	0.78	0.52	1.18
<40,000	291	28.9	235	29.9	56	25.3		0.66	0.42	1.05
Bicarbonate (HCO_3_)										
≥22	762	79.1	623	82.8	139	65.9	<0.001	1		
<22	201	20.9	129	17.2	72	34.1		2.5	1.77	3.52
Creatinine, mg/dL										
≤1.3	821	87.4	666	92	155	73.5	<0.001	1		
>1.3	114	12.2	58	8	56	26.5		4.15	2.76	6.23
Troponin levels, ng/ml										
≤0.04	93	68.9	59	84.3	34	52.3	<0.001	1		
>0.04	42	31.3	11	15.7	31	47.7		4.89	2.18	10.96
Pleural effusion										
No	792	78.6	57	8.5	34	16	0.002	1		
Yes	91	9	614	91.5	178	84		2.06	1.3	3.25
Acute cholecystitis										
No	872	98.8	666	99.3	206	97.2	0.02	1		
Yes	11	1.2	5	0.7	6	2.8		3.88	1.17	12.84
Echo findings										
Normal	987	97.9	783	99.5	204	92.3	<0.001	1		
Any cardiac pathology	21	2.1	4	0.5	17	7.7		16.31	5.43	49.01
Discharge mode										
Discharged	937	93	736	93.5	201	91	0.004			
LAMA	55	5.5	44	5.6	11	5		NA		
Death	16	1.6	7	0.9	9	4.1				

Characteristics of dengue patients by the length of stay in the hospital

We found that a higher proportion of the patients who stayed for more than three days (58.8%) were older than 35 years when compared with 54.4% of the patients who stayed for three days at most. Similarly, among diabetics, 14.5% of the patients stayed for more than three days as compared to 9.8% of their counterparts (p-value: <0.04). A significantly higher proportion of patients who stayed for more than three days (24.4%) were hypertensive compared with patients who stayed for three days at most (9.0%; p-value: <0.001).

About one-third of the patients who stayed for more than three days had hyponatremia when compared with 27.7% of patients stayed for three days at most (p-value: <0.03), as shown in Table 1. Around one-third of the patients (34.1%) who stayed for more than three days had lower levels of HCO_3_ as opposed to 17.2% of the patients who stayed for less than three days. A significantly higher proportion of the patients (47.7%) who stayed for more than three days had higher levels of troponin as opposed to 15.7% of the patients who stayed for three days at most. However, there were no significant differences between patients who stayed for more than three days and their counterparts who did not regarding levels of potassium, platelet count, and gender.

Factors associated with the length of stay in the hospital among dengue patients: findings of bivariate analysis

We found that dengue hyponatremia patients were 41% more likely to stay in the hospital beyond three days when compared to patients with normal levels of sodium (OR = 1.41; 95% CI: 1.03-9.35) (Table 1). Likewise, dengue patients who were diabetic were 1.56 times more likely to stay in the hospital beyond three days when compared to non-diabetic dengue patients (OR = 1.56; 95% CI: 1.01-2.43). Similarly, hypertensive patients were 3.26 times more likely to stay in the hospital beyond three days when compared to non-hypertensive patients (OR = 3.26; 95% CI: 2.20-4.83).

Furthermore, dengue patients with higher troponin levels were 4.89 times more likely to stay in the hospital beyond three days when compared with patients having normal values of troponin (OR = 4.89; 95% CI: 2.18-10.96). Patients with effusion were 2.06 times likely to stay in the hospital beyond three days when compared to patients without effusion (OR = 2.06; 95% CI: 1.30-3.25) (Table 1). On the other hand, factors such as hypokalemia and thrombocytopenia, patient age, and gender were not found to be significantly associated with the length of stay in the hospital beyond three days.

Association of hyponatremia with the length of stay in the hospital among dengue patients: findings of multivariable analysis

The results of the multivariable analysis were adjusted for patients' age, gender, troponin levels, effusion, platelet count, and comorbidities such as DM and HTN. The results demonstrated that associations between hyponatremia (aOR = 1.29; 95% CI: 0.59-2.84), hypokalemia (aOR = 2.36; 95% CI: 0.91-6.10), DM (aOR = 0.84; 95% CI: 0.34-2.07), effusion (aOR = 1.37; 95% CI: 0.52-3.66) and patient’s stay in the hospital beyond three days disappeared in the final adjusted model after controlling for potential confounders. However, the association between troponin levels (aOR = 5.74; 95% CI: 2.34-14.11) and patient’s stay in the hospital beyond three days persisted in the adjusted model and became stronger relative to the bivariate analysis, as shown in Table 2.

**Table 2:** Association between electrolyte imbalances and morbidity and mortality in dengue-infected patients admitted to a tertiary care hospital (n=1,008) The model for length of stay is adjusted for age, gender, diabetes mellitus, troponin, and effusion. The model for mortality is adjusted for age, gender, diabetes mellitus, creatinine, and effusion aOR: adjusted odds ratio; CI: confidence interval

	Length of stay for more than three days			Mortality		
	aOR	95% CI		aOR	95% CI	
Age, years						
≤35	1			1		
>35	0.84	0.3	2.31	0.43	0.12	1.54
Gender						
Male	1			1		
Female	0.84	0.37	1.87	1.67	0.51	5.55
Diabetes mellitus						
No	1			1		
Yes	0.84	0.34	2.07	4.36	1.21	15.74
Sodium levels, meq/L						
≥135	1			1		
<135	1.09	0.49	2.38	1.4	0.44	4.49
Potassium levels, meq/L						
≥3.5	1			1		
<3.5	2.36	0.91	6.1	0.29	0.04	2.43
Creatinine, mg/dL						
≤1.3		NA		1		
>1.3				14.738	4.19	51.85
Troponin levels, ng/ml						
≤0.04	1					
>0.04	5.75	2.34	14.11	NA		
Effusion						
No	1			1		
Yes	1.37	0.52	3.66	2.35	0.64	8.64

Association of hyponatremia with mortality among dengue patients: findings of multivariate analysis

The results of the multivariable analysis were adjusted for the patients' age, gender, creatinine levels, pleural effusion, platelet count, and comorbidities such as DM and HTN. The results demonstrated that there was no statistically significant association of electrolyte imbalances such as hyponatremia (aOR = 1.40; 95% CI: 0.44-4.49), hypokalemia (aOR = 0.29; 95% CI: 0.03-2.43), and effusion (aOR = 2.35; 95% CI: 0.64-8.64) with mortality in the final adjusted model after controlling for potential confounders. However, there was a statistically significant association of creatinine levels (aOR = 14.74; 95% CI: 4.19-15.85) and DM (aOR = 4.36; 95% CI: 1.21-15.74) with mortality after controlling for potential confounders and it persisted in the adjusted model; however, 95% CI for these associations were not precise as shown in Table 2.

## Discussion

We undertook this study to assess the association of electrolyte imbalance (hypokalemia and hyponatremia) with the length of stay and mortality in dengue-infected patients. In the absence of overt complications, dengue-infected patients are usually discharged within three days of hospitalization. Our study showed that 78.0% had less than three days of stay in the hospital with a mean length of stay of 2.58 days. Most of the national and international literature have reported similar results [11,12]. After controlling for potential confounders, we did not find any statistically significant association of electrolyte imbalances such as hyponatremia and hypokalemia with the length of stay and mortality. The possible explanation for these null findings could be that hospitalized patient get fluid therapy and their electrolytes are rapidly corrected. Moreover, we did not find any association of age, gender, electrolyte disturbances, and metabolic acidosis at the time of admission with either the length of stay or case fatalities.

We found that at the time of admission, almost one-third of the patients had electrolyte imbalance, i.e, hyponatremia (29.3%) and hypokalemia (23.2%), and a similar proportion of the patients had lower levels of HCO_3_. Incidentally, Joshi et al. have established the occurrence of hyponatremia to be 40.3% in dengue patients [13], which is close to the prevalence of hyponatremia in our study population, while another study of pediatric patients in Thailand has shown the prevalence of hyponatremia to be 61%, which went up to 72% with increased disease severity. The incidence of hypokalemia was noticed to be 14-17% as compared to 23% in our study. The main reason for the incidence of hyponatremia was presumed to be amplified antidiuretics hormone secretion (ADH) while hypokalemia was attributed to renin-angiotensin-aldosterone activation resulting in the loss of potassium in the urine [14]. Nevertheless, electrolyte imbalances failed to show any impact on either the length of stay or mortality.

Critical organ involvement is not uncommon in dengue infection; myocarditis is known to occur in dengue and is diagnosed either by clinical sign and symptoms or elevated cardiac markers including troponin and creatine kinase isoenzyme MB (CK-MB). Arora et al. found elevated troponin in 23.3% of their cohort, but all those patients had normal echocardiography [15], while we found it to be elevated in almost one half (31.3%) of our study population, though it did not have any impact on mortality; however, a higher proportion of these patients had longer stay in the hospital when compared to those who had normal levels of troponin (47.7% vs. 15.7%).

In our study, we found that around more than half of the patients (55.4%) were older than 35 years, which is consistent with earlier reports from the southern part of the country [16]. In the hyperendemic areas of India, a large cohort of test-positive dengue patients had a median age of 25 years [interquartile range (IQR) = 16-36 years] [17]. A recent outbreak report from the northern areas of Pakistan showed similar age group distribution [18]. These variations of age across the region can be attributed to data collection patterns. We noted that 58.8% of patients who stayed for more than three days in the hospital were older than 35 years, which may be attributed to the fact that patients in higher age groups generally have a longer duration of hospital stay. Such a pattern is attributed to many factors such as patients in older age groups being more prone to infections secondary to weak immune responses, their having more comorbid conditions, and prolonged exposure to environmental hazards such as pollution and contaminated water. Moreover, Choudhary et al. have validated the correlation of patients of a higher age group with a severe form of dengue infection [19].

Our study showed 68.4% male preponderance among the cohort, a known pattern seen in South Asian populations. Our data is similar to many reports from Pakistan, India, and Bangladesh [20,21]. On the contrary, studies from South American regions have shown that females are more infected as opposed to males [12,21]. Since mosquitoes are gender-blind, any gender association seems more likely due to the cultural differences among different nations. For example, Ali et al. found no association of DVI with gender [11]. These differences may be attributed to the fact that men are more exposed to the outside environment as compared to females in Pakistan, where women are generally more involved in-home activities and many of them observe Purdah/Hijab (covering themselves completely) when going outside owing to their religious and cultural beliefs. Also, reduced access to health services means that hospital-derived data might have missed cases of mild and moderate nature and might have over-represented severely affected diseases. In Pakistan, where diabetes has reached endemic proportions with a prevalence of 26.8%, regular seasonal surges of DVI can make most of the infected population vulnerable to severe disease. In our study cohort, diabetic patients were more likely to stay in the hospital beyond three days in the univariable model. Moreover, diabetic patients had a higher risk of mortality in the multivariable model, as compared to non-diabetic patients infected with dengue. Investigators have observed that DM could increase the severity of thrombocytopenia in dengue-infected patients [22]. Similar findings were noted in a systematic review of literature where a severe form of DVI was noticed in patients with DM and HTN [23,24]. This phenomenon may be due to DM being associated with decreased host immunity to infections via decreased functioning of immune cells, including neutrophils, macrophages, T cells, and antibody-producing B cells [25].

Our study revealed that 11.8% of patients had elevated serum creatinine levels at the time of admission, which considerably influenced the in-hospital mortality. Renal Injury is a known phenomenon in dengue patients, which can be a direct viral cytopathic effect on renal tubular cells to, more commonly, hemodynamic instability leading to hypoperfusion injury and a less common behavior like rhabdomyolysis and immune-mediated direct or indirect complex injuries [26]. The incidence of acute kidney injury (AKI) in DVI has shown incongruity, where data mostly originated from observational retrospective studies, and reported a range of 0.83-14.40% with a mortality rate of 11.30-60.00%.

Our study findings need to be interpreted in light of some limitations. We collected retrospective data of biochemical parameters from in-hospital records. In limited-resource regions, more than half of the patients are usually missed out in the studies since data relies on in-hospital admissions. We were unable to grade the severity of the disease due to coding limitations; grading the severity might have helped us in predicting the disease activity. This study was limited to the adult population, hence inferences cannot be extrapolated to patients in pediatric age groups, which account for a substantial percentage of the disease incidence. Since this study was only conducted in one hospital, findings cannot be generalized to larger communities of Pakistan or neighboring countries. Besides, we did not collect the data on the management of electrolyte imbalance, such as intravenous fluid therapy, which could also confound the association between electrolyte imbalance and the length of stay. Moreover, we could not assess the temporal association between various predictors of dengue and the length of stay. Thus, more robust epidemiological studies are required in the future to assess the association between electrolyte imbalance and the length of hospital stay in dengue patients.

## Conclusions

To recapitulate, our study found that myocarditis, as evidenced by elevated troponin levels, had a significant impact on the length of stay in dengue patients, whereas AKI and DM influenced the outcomes in terms of mortality. This study provides the basis of evidence for mortality predictors in a high-risk population where comorbidities and non-communicable diseases are both endemic. Large epidemiological and robust studies are required to assess the association between electrolyte imbalance and the length of stay across different areas of Pakistan, which could help device preventive measures and in-hospital admission pathways to mitigate dengue-associated complications.

## References

[1] Shivanthan MC, Rajapakse S (2014). Dengue and calcium. Int J Crit Illn Inj Sci.

[2] Vachvanichsanong P, Thisyakorn U, Thisyakorn C (2016). Dengue hemorrhagic fever and the kidney. Arch Virol.

[3] (2020). WHO: Global strategy for dengue prevention and control 2012-2020. https://apps.who.int/iris/bitstream/handle/10665/75303/9789241504034_eng.pdf.

[4] Nguyen MT, Ho TN, Nguyen VV (2017). An evidence-based algorithm for early prognosis of severe dengue in the outpatient setting. Clin Infect Dis.

[5] Iqbal SP, Jaffri SA (2020). Dengue: a recent challenge in Pakistan. J Bahria Univ Med Dent Coll.

[6] Fatima Z (2019). Dengue infection in Pakistan: not an isolated problem. Lancet Infect Dis.

[7] (2020). WHO guidelines approved by the Guidelines Review Committee. Dengue: Guidelines for Diagnosis, Treatment, Prevention and Control: New Edition. World Health Organization. https://apps.who.int/iris/bitstream/handle/10665/204894/B4751.pdf.

[8] Vikram K, Nagpal BN, Pande V (2016). An epidemiological study of dengue in Delhi, India. Acta Trop.

[9] Maheshwari M, Bansal R, Gupta S (2017). Electrolyte profile of dengue infected patients: an observational study from a tertiary care centre in Rajasthan. Indian Pract.

[10] Vachvanichsanong P, McNeil E (2015). Electrolyte disturbance and kidney dysfunction in dengue viral infection. Southeast Asian J Trop Med Public Health.

[11] Joshi R, Baid V (2011). Profile of dengue patients admitted to a tertiary care hospital in Mumbai. Turk J Pediatr.

[12] Lumpaopong A, Kaewplang P, Watanaveeradej V (2010). Electrolyte disturbances and abnormal urine analysis in children with dengue infection. Southeast Asian J Trop Med Public Health.

[13] Arora M, Patil RS (2016). Cardiac manifestation in dengue fever. J Assoc Physicians India.

[14] Khalil A, Qurash M, Saleh A, Ali R, Elwakil M (2016). Multiple renal abscesses due to ESBL extended-spectrum beta-lactamase-producing Escherichia coli causing acute pyelonephritis and bacteremia: a case report with a good outcome (no drainage required). Case Rep Infect Dis.

[15] Murhekar M, Joshua V, Kanagasabai K (2019). Epidemiology of dengue fever in India, based on laboratory surveillance data, 2014-2017. Int J Infect Dis.

[16] Abdullah Abdullah, Ali S, Salman M (2019). Dengue outbreaks in Khyber Pakhtunkhwa (KPK), Pakistan in 2017: an Integrated Disease Surveillance and Response System (IDSRS)-based report. Pol J Microbiol.

[17] Chaudhry M, Ahmad S, Rashid HB, Ud Din I (2017). Dengue epidemic in postconflict Swat District, Khyber Pakhtunkhwa, Pakistan, 2013. Am J Trop Med Hyg.

[18] Khalil MA, Tan J, Khalil MA, Awan S, Rangasami M (2014). Predictors of hospital stay and mortality in dengue virus infection-experience from Aga Khan University Hospital Pakistan. BMC Res Notes.

[19] Agarwal R, Kapoor S, Nagar R (1999). A clinical study of the patients with dengue hemorrhagic fever during the epidemic of 1996 at Lucknow, India. Southeast Asian J Trop Med Public Health.

[20] Guha-Sapir D, Schimmer B (2005). Dengue fever: new paradigms for a changing epidemiology. Emerg Themes Epidemiol.

[21] Ali A, Rehman HU, Nisar M (2013). Seroepidemiology of dengue fever in Khyber Pakhtunkhawa, Pakistan. Int J Infect Dis.

[22] Chuang TW, Ng KC, Nguyen TL, Chaves LF (2018). Epidemiological characteristics and space-time analysis of the 2015 dengue outbreak in the metropolitan region of Tainan city, Taiwan. Int J Environ Res Public Health.

[23] Toledo J, George L, Martinez E (2016). Relevance of non-communicable comorbidities for the development of the severe forms of dengue: a systematic literature review. PLoS Negl Trop Dis.

[24] Lee IK, Hsieh CJ, Lee CT, Liu JW (2020). Diabetic patients suffering dengue are at risk for development of dengue shock syndrome/severe dengue: emphasizing the impacts of co-existing comorbidity(ies) and glycemic control on dengue severity. J Microbiol Immunol Infect.

[25] van Crevel R, van de Vijver S, Moore DAJ (2017). The global diabetes epidemic: what does it mean for infectious diseases in tropical countries?. Lancet Diabetes Endocrinol.

[26] Lim CT, Fuah KW, Lee SE, Kaniappan K, Fah TR (2019). Dengue-associated acute kidney infection: an updated and comprehensive qualitative review of literature. Nephrology.

